# A Systematic Review of Culturally Responsive Approaches to Child and Adolescent Mental Health Care in Ethiopia

**DOI:** 10.3389/fsoc.2020.583864

**Published:** 2021-02-02

**Authors:** Hana Shewamoltot Meshesha, Veronica Johnson

**Affiliations:** Department of Counseling, University of Montana, Missoula, MT, United States

**Keywords:** wellness approach, pyramid model, child and adolescent mental health, Ethiopia, culturally responsive

## Abstract

In 2012/2013, the prevalence of child mental illness was estimated to be between 12 and 25% in Ethiopia. The Federal Ministry of Health is currently considering the implementation of the second national mental health strategy guided by the World Health Organization’s pyramid model for an optimal mix of services. This model states self-care as the fundamental concept and practice that can be facilitated by the formal and informal sectors surrounding an individual. Despite this remarkable move toward endorsing contemporary approaches to mental health services, Ethiopia struggles with a limited number of knowledgeable and skilled mental health professionals. This systematic review aims at identifying the main challenges Ethiopia might face while implementing the pyramid model. We will suggest ways to bridge the gap between the need for child/adolescent trained mental health professionals and training mental health professionals informed with the value of integrating the concepts of the pyramid model into the system of mental health care for Ethiopians. The paper also discusses the importance of integrating the Wellness based mental health approach into mental health professionals’ training as a means of developing a culturally responsive approach to child and adolescent services. This review provides implications for further studies and mental health policy, systems and services in Ethiopia.

## Introduction

Ethiopia developed its first National Mental Health Strategy (NMHS) in 2012/2013. This NMHS was an initiative by the Federal Ministry of Health (FMOH) intended to be implemented within 5 years (Federal Ministry of Health, 2012: 1–4). The strategy was initiated following the assessment of the barriers for achieving the Millennium Developmental Goals (MDGs). The strategy recognizes the relevance of well-being focused mental health services and specifies activities in line with the five-tiers of the World Health Organization (WHO) pyramid of mix of mental health services (Federal Ministry of Health, 2012: 18). In the document, the FMOH stipulates the prevalence of major mental health issues in Ethiopia. Specifically, it reported that the prevalence of childhood mental illness ranges from 12 to 25% (information on types of mental health disorders was not provided). Furthermore, the prevalence of completed and attempted suicide rates was stated as 7.7/100,000/year and 3.2% respectively, although, no specific demographic information was provided to better understand the prevalence among children and adolescents (Federal Ministry of Health, 2012: 11). Based on data collected in 2016, the World Health Organization (2019: 19) reported the age-standardized suicide rate in Ethiopia was 11.4 with males having a higher rate (18.7) compared to females (4.7). The World Health Organization (2019, 11) global report states that suicide rates are higher at younger ages—adolescents living in low-income and middle-income countries constituting the majority of deaths by suicide. These prevalence rates demand attention in an effort to build a comprehensive mental health system for Ethiopians.

In 2019, as part of a mental health forum, the FMOH identified more specific categories of mental health problems among children and adolescents on its website, i.e., ADHD, oppositional defiant disorders/conduct disorders, anxiety disorders, mood disorders, elimination disorders, and autism spectrum disorders, with prevalence rates of 12–25%.The report showed an increment in suicide rates and the prevalence of alcohol and substance use disorders across a 10-year span. The report also showed a 21.56–27.9% prevalence for “common mental health disorders” (depression, anxiety and psychological distress) by referring to a meta-analysis [author(s) unspecified] conducted in 2018 (http://www.moh.gov.et/ejcc/am/node/170). However, the report did not include information on specific demographic variables. Although FMOH did not portray its data against the prevalence rates of mental disorders in the other countries, based on a report published in World Health Organization (2017b), the WHO stated that about 20% of children and adolescents experience mental health problems globally (https://www.who.int/health-topics/mental-health#tab=tab_2). The lack of specificity in the data provided by FMOH could be a deterrent in the implementation of the NMHS as well as the provision of preventative and mental health care treatment for Ethiopians.

According to a study by Hanlon et al. (2017: 8–9), there is a great deal of support at the national leadership level for mental health services in Ethiopia. However, governance at the grassroots level, among primary health care workers, is not to the level that meets the needs of the community. In 2017a, in its members’ status report, the WHO reported low numbers of mental health professionals in Ethiopia in comparison to the total population and to the prevalence of mental health problems (World Health Organization, 2017a: 1). The report indicated a total of 1,739 mental health professionals were working in both governmental and non-governmental sectors at the time. The rate of psychiatrists per 100,000 was 0.08% and the rate for mental health nurses was 1%. The rate of other mental health professionals such as psychologists, social workers and other paid mental health workers accounted for 0.04, 0.04, and 0.54%, respectively (World Health Organization, 2017a: 1). Addressing the mental health needs of Ethiopians requires the availability of a knowledgeable and skilled human resource. The integration of mental health services into the mental health system also needs to be culturally sensitive to the individuals in need for a successful outcome.

Several studies related to mental health issues in Ethiopia focus on the role of the intersectionality of client identities into the mental health services provided (e.g., Ghebrehiwet et al., 2019; Chisholm et al., 2020). There are also a number of studies emphasizing causal factors, challenges and successes in service provision for mental health services (e.g., Hambisa et al., 2020). However, there few studies conducted focusing on the preparedness and/or the training of mental health professionals from a culturally responsive perspective. The studies conducted to assess the challenges faced by mental health professionals in providing for Ethiopians in need are also limited (e.g., Hanlon et al., 2017; Wondie and Abawa, 2019; Zeleke et al., 2019). This systematic review was intended to suggest a culturally responsive approach to mental health services for children and adolescents in Ethiopia through building the capacity of mental health professionals. The aim of this review is to promote discussion about the systemic approaches and strategies for mental health services in Ethiopia. First, it is important to address some of the unique aspects of Ethiopian culture that provide context and rationale for a specific strategy to address the mental health needs of children and adolescents in Ethiopia.

The perceptions of Ethiopians regarding the causes of mental disorders and individuals’ preferred treatment options play a significant role for how the formal and informal mental health care system interact. For instance, Abera et al. (2015: 6) reported that a significant percentage of parents mentioned supernatural powers (e.g., evil spirits, God’s will, curse…) causing mental disorders among children and adolescents. In this study, parents reported that they would prefer to seek religious and traditional mental illness treatment modalities as opposed to formal health care. Abera et al. (2015: 7) also revealed that the majority of parents recognize the role of genetic and environmental factors (e.g., family financial problems, divorce, and abuse) as causal factors for child/adolescent mental illness. Recognizing mental health symptoms at a microsystem level plays a significant role in early detection and provision of treatment of mental disorders among children/adolescents. According to some studies, Abera et al. (2015: 7) and Kerebih et al. (2018: 6), parents and teachers recognize mental disorders with externalized behaviors compared to those with internalized behaviors. In addition, although externalized behaviors are recognized, parents and teachers do not associate the behaviors with mental disorders and seek professional support.

The above studies show that the perception of mental health in Ethiopia is guided by spiritual beliefs that seem to associate biological and environmental factors with the causes of mental illness. As a result, the effective implementation of a mental health strategy needs to bring the informal sector to the table for a shared meaning and vision to be created among Ethiopians and to serve those in need. The mental health system in Ethiopia, guided by the NMHS through the WHO comprehensive pyramid model, could be supported by integrating the wellness approach to mental health. The wellness model emphasizes individual wellbeing as a focus of mental health care providing opportunities for professionals to integrate cultural, spiritual, biological and environmental factors contributing to mental disorders.

## Materials and Methods

We conducted a literature review on mental health in Ethiopia, using Google, Google Scholar and PsychINFO search engines and websites of organizations such as the WHO, FMOH, and Federal Ministry of Education (FMOE). In the literature identification process, for research articles, the search criteria included: research conducted in Ethiopia, research focused on integrating cultural factors into the mental health service provision, mental health among children and adolescents, research that has a framework or a systemic approach to addressing mental health issues and services in Ethiopia, and research studies integrating the concept of a wellness approach. For research articles, studies published between 2014 and 2020 were included. This time period was chosen to include studies conducted following the implementation of the first NMHS in 2012/2013. Research articles were excluded if the study was focused on clinical trials, was non-peer reviewed and focused on non-systemic or framework-oriented research. For other framework and approach-oriented publications, the publication dates were considered between 2000 and 2020. Publications included under the “other framework and approach-oriented publications” were specific to the WHO’s Pyramid model, the Wellness Model and the Indivisible Self-Model. For fundamental conceptual paradigms (e.g., concept of wellness, the WHO’s Pyramid model), original publications were reviewed and their publication year dated back to 2000. During the identification, 28 research articles and 21 other publications were identified. Of these, three articles and five other publications were removed due to duplication.

During the screening process, research studies were included that focused on systemic reviews of the mental health systems and policy in Ethiopia, training of mental health professionals, and expanding/alternative approaches to mental health services. Research studies that focused on causes of mental illness, mental health disorders and their consequences, mental health training for professionals, child trafficking, food insecurity and mental health, mental health among women, mental health among college students, and mental health in specific ethnic, economic and ways of life were excluded. For other publications, contents that did not provide general guidance on the framework and approaches to mental health were excluded. Accordingly, 12 research studies and 20 other publications were selected. Of these, 27 articles and other publications were chosen for inclusion in this review.

The eligibility process for selecting articles and publications emphasized the first NMHS and the WHO’s pyramid model of optimal mix of mental health services as guiding mental health service approaches for an Ethiopian context. These documents were chosen to be the focus for the following reasons. First, the NMHS was the first of its kind that Ethiopians developed to tackle the barriers identified during the implementation of MDGs. As a result, it provided information on national level directions. Second, the NMHS emphasizes the role of the WHO’s pyramid model of optimal mix of mental health services as a guiding strategy to organize national and local level mental health services. The model is used as a reference for training community-based mental health professionals in Ethiopia. As a result, the pyramid model allows a review of the training of mental health professionals according to the mental health service approach endorsed by FMOH. Third, the emphasis of the WHO’s pyramid model of optimal mix of mental health services on self-care and informal care systems allows the integration of culturally responsive mental health services. As a result, nine articles and 10 other publications were included in the qualitative data synthesis.

## Results

### Systematic Reviews and Meta-Analyses

This systematic review of relevant and timely publications illustrates the WHO’s pyramid model of optimal mix of mental health services, the mental health profile of Ethiopia, mental health professionals practicing in Ethiopia, and ways to bridge the gap between the need for mental health services and integration of a comprehensive approach to such services. The following table shows the type of articles and publications used in the synthesis of this review and the common themes that emerged.

In Table 1, the common themes that emerged included cultural factors that researchers indicated either as barriers to the implementation of effective mental health services or indicated as relevant factors to be considered in mental health services. The themes also included organizational, theory and approach-based recommendations for mental health services. The training, challenges and success of mental health services were identified. These common themes were further organized as culture and mental health in Ethiopia, WHO’s model for comprehensive mental health services, mental health professionals in Ethiopia, bridging the gap: the wellness model of mental health, on-the-job training for school-based professionals and adoption of child/adolescent specific mental health initiatives.

**TABLE 1:** Description of studies included in this review.

Author(s) (Year of publication)	Type of publications	Topic/variables	Main themes of publications
Ahmed et al. (2019)	Original research	Mental illness service provision and associated factors	Knowledge, attitude, and practice among health extension professionals
Adler [(1927) 1954]	Theory	Individual psychology	Wholistic approach to mental health
Assefa et al. (2019)	Systemic review and synthesis	Community health extension program of Ethiopia	Challenges for mental health professionals
Desta et al. (2017)	Original research	Empowering preschool teachers to identify mental health problems	Integrating mental health services in schools
Faregh et al. (2019)	Original reflection and overview	Considering culture, context and community in mhGAP implementation and training	Field perspective on *mhGAP trainings*
FMOE (2019)	Annual report	National annual enrollment	Percent of children and adolescents in schools
FMOH (2012)	Strategy document	National mental health strategy	Mental health profile
			Challenges to mental health services
			The optimal mix of mental health services
Kebede (2019)	Original research	Social work education in Ethiopia	Expanding mental health services
Myers and Sweeney (2004) and Myers and Sweeney (2008)	Original research	Wellness model	Wellness based approach
		The indivisible self-model	Wellness wheel
Sixty-Sixth World Health Assembly (2013)	Meeting minute	Comprehensive mental health action plan	Conceptualizing mental health
WHO (2003a)	Policy and service guidance	Caring for children and adolescents with mental disorders: Setting WHO directions	The optimal mix of mental health services
WHO (2003b)	Policy and service guidance	Organization of services for mental health: Mental health policy and service guidance package	The optimal mix of mental health services
WHO (2009)	Guidance to improve mental health services	*Improving Health Systems and Services for Mental Health*	Cultural integration into mental health services
WHO (2016a)	Training manuals	*mhGAP* training of trainers manual	Guide for training of trainers of community health workers
WHO (2016b)	Training manuals	*mhGAP Intervention Guide*	Training of community health workers
WHO (2017a)	Report	Mental health profile of Ethiopia	Profile of mental health professionals
			Existence of strategy and plan
Wondie (2014)	Reflection	Development of psychology in Ethiopia and future directions	Overview of mental health training fields in Ethiopia
Wondie and Abawa (2019)	Original research	Westernization vs. indigenization	Lack of utilizing indigenous cultural resources in mental health services
Zeleke et al. (2019)	Original research	Counseling alignment of culture	Challenges for mental health professionals
			Cultural factors
			Wellness approach

### Culture and Mental Health in Ethiopia

In multiple mental health status reports, it is common to see the lack of specific information on Ethiopia’s systems of assessment and intervention for mental health disorders. The NMHS states cultural factors as reasons contributing to the inability to gather accurate data on the prevalence of mental health problems and providing professional services (Federal Ministry of Health, 2012: 12). Specifically, the challenge is associated with the fact that many Ethiopians associate severe mental illness such as schizophrenia and mood disorders with spiritual causes. This data is also supported by Zeleke and colleagues’ (2019: 224) study suggesting that “cultural myths” about the source of mental illness as a curse present challenges for mental health professionals in their counseling practice. Moreover, mental health issues like depression and anxiety are often managed in informal relationships and spiritual-based care (Federal Ministry of Health, 2012: 12). Individuals commonly decide to seek treatment from religious leaders and healers rather than seeking professional support. This has resulted in fewer opportunities for early detection of depressive symptoms that might have resulted in support for individuals with suicidal ideation (Federal Ministry of Health, 2012: 12). Furthermore, the lack of mental health professionals at primary health care facilities adds to the difficulty of obtaining accurate early identified symptoms. Therefore, to tackle the problem, national goals such as adopting the pyramid model of optimal mix of mental health services to focus on patient-based care, community involvement, integrating mental health services in health extension programs, monitoring and evaluation, and collaborating with higher education institutions that prepare mental health professionals were emphasized (Federal Ministry of Health, 2012: 3–4).

After the implementation of the first NMHS, Ethiopia is currently working on the second NMHS, with the intent of incorporating the successes obtained and lessons learned during the implementation of the first NMHS (http://www.moh.gov.et/ejcc/am/node/170). Moreover, FMOH, through the NMHS, is still using the WHO’s pyramid model for a combination of services contributing to quality mental health services (Federal Ministry of Health, 2012: 18). In its forum on mental health situations in Ethiopia, a presentation entitled *Strategies for Promotion and Prevention and Rehabilitation in Mental Health in Ethiopia* included self-care as the fundamental component of mental health based on the pyramid model (http://www.moh.gov.et/ejcc/am/node/171). The mental health wellbeing approach as a framework for future mental health initiatives and services in Ethiopia was also addressed.

### The WHO’s Model for Comprehensive Mental Health Services

Providing quality mental health services requires the integration of multiple levels of services involving the individual, informal care and formal care services. The World Health Organization (2003b: 34) identified a model that can be used as a framework for coordination of mental health services, and also as a guide or framework for member countries to improve the mental health care in their specific cultural context. While countries may choose to design their own respective comprehensive models of mental health services, the pyramid model of optimal mix of mental health services underlines the basic components that help services to be accessible to individuals in need. In 2009, the WHO stated: “there is no single organizational model for good service delivery, there are common factors that underlie successful approaches” (World Health Organization, 2009: 21).

In Figure 2, self-care is identified as the foundation for other formal mental health care services. The awareness and intentional use and modeling of self-care strategies by an individual could result in less time in mental health facilities which, in turn, becomes less costly for both the individual and the nation. The availability and integration of informal community care, such as neighborhood-based self-help groups, faith-based groups for children and adolescents, neighborhood gatherings and other informal regular group events designed for celebrations and support could enhance individuals’ perception of acceptance and care while they are taking care of themselves. In this case, the role of formal health care can be providing information to raise awareness about preventive strategies for mental illness, such as educating parents how to recognize mental health issues and implementing strength-based approaches to help their children. Parents/caregivers can be taught how and when to seek professional help and what their role is in supporting the intervention provided by mental health professionals. Neighbors, faith-based group leaders and self-help group leaders can be trained in strength-based community support strategies to help children and adolescents in their communities. Stakeholders in the informal care system can also impart their rich knowledge and experience to the formal system by indicating how the community perceives mental health, what symptoms represent what mental disorders, how traditional treatment (e.g., spiritual based practices) can support the formal system, and so on. The informal care system can be used as an ally to support the formal professional service with evidence-based culturally sensitive education. In addition, the informal care system can be best utilized by integrating mental health education that integrates wellness including spiritual wellbeing. The WHO’s model for mix of mental health services addresses the well-being of individuals with acknowledgment of cultural norms and values around the person. The focus on self-care as an underlying component also provides a foundation for integrating personal and cultural identities as well as an opportunity to address the intersectionality of the two factors.

**FIGURE 1:**
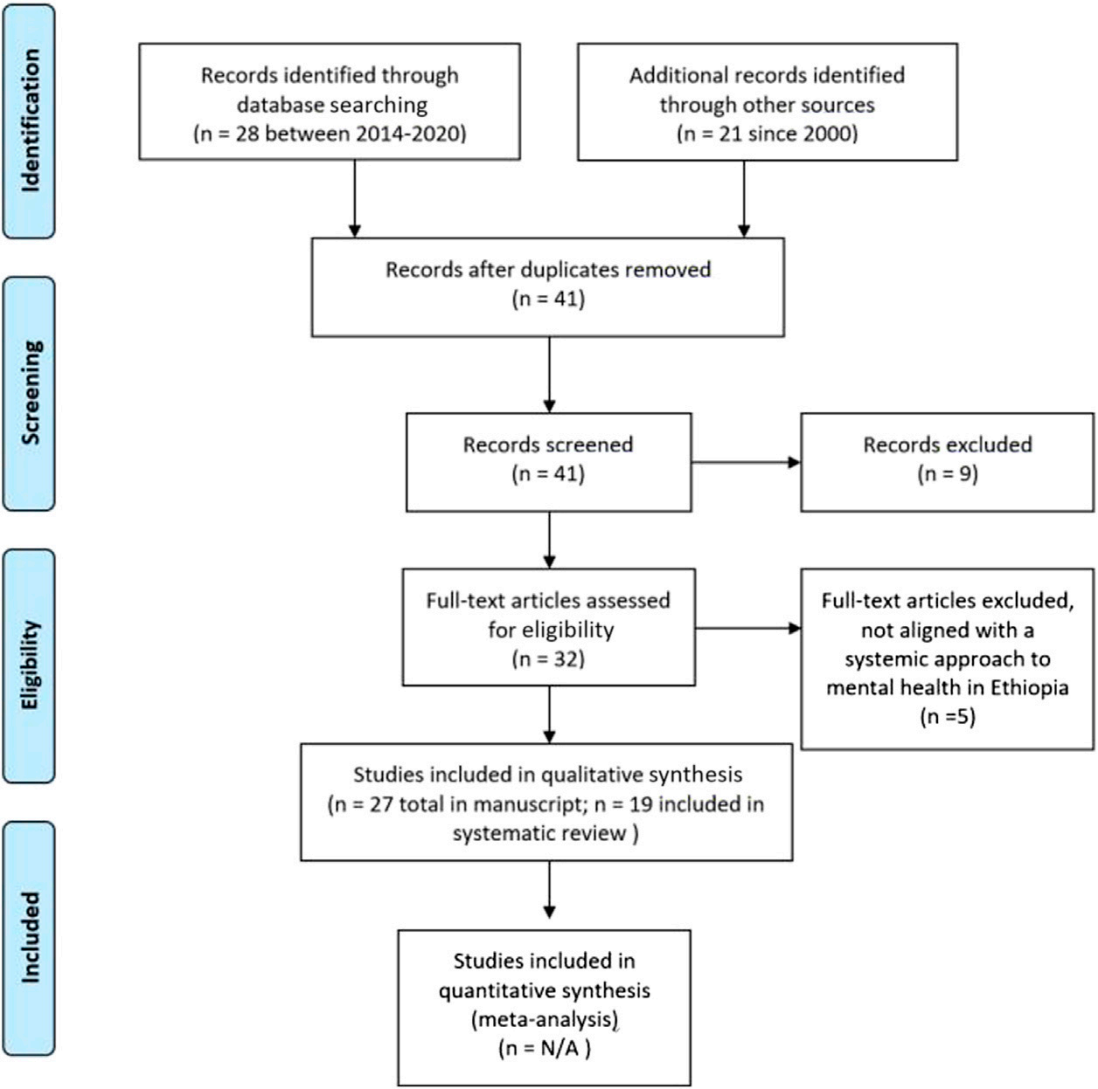
Literature identification procedure (Moher et al., 2009: 3).

**FIGURE 2:**
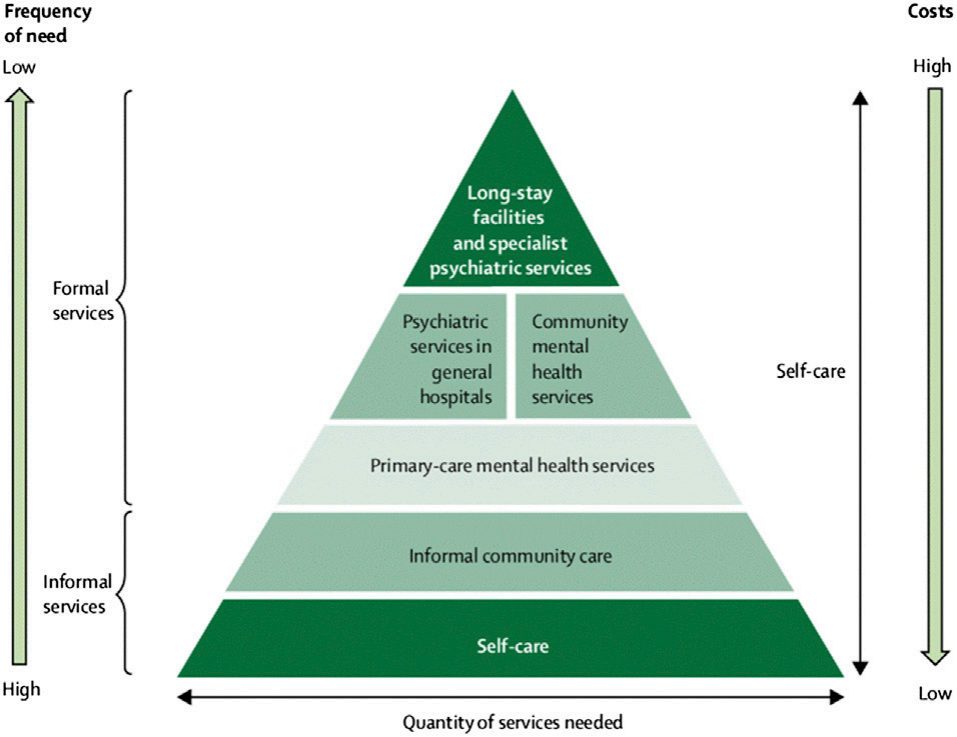
WHO service organization pyramid model for comprehensive services in mental health.

In its guidelines for caring for children and adolescents with mental health issues, the World Health Organization (2003a: 7) indicates that diagnosis of mental health disorders should not be done based on just one cultural perspective. It is essential to integrate the relevant culture-based information in assessment and diagnostic processes. The guideline also indicates the need to effectively utilize resources that are immediate to the child or adolescent in need, i.e., family members, neighbors and religious leaders for both efficacy and efficiency. Furthermore, the WHO stresses the need to adopt a wellness-based approach to managing mental health issues, specifically when helping children and adolescents. The WHO’s 5-year mental health action plan is in place until the end of 2020. Within the action plan, the World Health Assembly states the following:

This action plan…. conceptualized mental health as a state of well-being in which the individual realizes his or her own abilities, ….With respect to children, an emphasis is placed on the developmental aspects, for instance, having a positive sense of identity, the ability to manage thoughts, emotions, and to build social relationships, as well as the aptitude to learn and acquire an education, ultimately enabling their full active participation in society (Sixty-Sixth World Health Assembly, 2013: 3).

In the above conceptualization, mental health intervention requires a well-rounded developmental approach for children. It addresses the holistic approach that needs to be included to ensure the well-being of children, to support them in achieving their full potential, and encouraging their participation in their society. In speaking of the mental health needs of children in Ethiopia, it is critical to review the system of mental health services for adults, as knowledge of mental health issues, access to mental health care, and implementation of effective treatment strategies all require the involvement and endorsement of adult caregivers.

Ethiopia, as stated in the NMHS, endorsed the pyramid model in its strategy and designed several activities to be implemented accordingly. The strategy includes specific goals designed to improve the decision-making ability of individuals, awareness-creating and skill-development trainings as goals at the informal care level and specific strategies at the primary health care levels. Most of these advocacy, awareness-raizing and skill-development activities were planned to be implemented by health extension workers after receiving training in the area (Federal Ministry of Health, 2012: 23–25). The integration of wellness-based approaches into mental health services requires orienting the health extension workers and mental health professionals to the approach and its implementation to meet the needs of individuals with mental health problems.

### Mental Health Professionals in Ethiopia

Professional mental health training for college-level social science students is a recent development in the formal educational system of Ethiopia. Modern psychology was introduced in the 1950s and its training focused on guidance and counseling and educational measurement (Wondie, 2014: 585). Later, other fields in psychology such as developmental psychology and social psychology were established in graduate level programs.

Along the educational expansion in Ethiopia, other mental health fields such as social work were established at the undergraduate and graduate levels in several universities across the country. Currently, most first-generation and second-generation public universities in Ethiopia have programs in psychology and/or social work. For instance, social work programs are available in 13 Ethiopian universities (K 2019: 5). Most first-generation universities that have undergraduate programs also have graduate level programs in those fields. However, it is not clear how many students graduated from those programs thus far. The lack of national data on the number of mental health graduates from Ethiopian universities and their placements creates a challenge in portraying the work of these professionals. The coordination of services between these mental health professionals and the formal health care system is not well known. It is unclear whether/how these mental health professionals are trained to familiarize themselves with either a wellbeing approach or the WHO’s model of optimal mix of mental health services. In our research, we were able identify that the undergraduate psychology curriculum provides the opportunity for students to learn about child and adolescent developmental milestones but does not address individual well-being as a concept or as an intervention strategy.

The WHO’s report depicts a clear picture of the lack of mental health professionals to address the mental health needs of the people in Ethiopia. The report only included one child psychiatrist for the whole country. Most of the outpatient mental health services report no specific categories of services offered or an annual policy/plan for development (World Health Organization, 2017a: 1). This clearly shows, on one hand, the obvious lack of professionals in the country and, on the other hand, the problems in efficiency of reporting systems. It seems that FMOH reports the number of mental health professionals working in medical facilities. This number is in contrast to the number of students graduating each year from universities in different helping professions who either decided not to (or who did not have the opportunity to) join the health care system. This suggests a lack of coordination between universities that train mental health professionals and existing mental health services in Ethiopia that serve adolescents and children with mental health issues. For instance, although the number of mental health workers in school settings might be low, it is worth reporting them as part of the mental health workforce. Both the WHO and NMHS did not report the mental health professionals working in schools. However, there are indications of the presence of mental health professionals in schools. These professionals are mostly first-degree psychology graduates, master’s graduates in social psychology and school psychology graduates primarily working with adolescents. School psychology is a program provided by one of the pioneer universities in Ethiopia, Bahir Dar University (https://bdu.edu.et/febs/). Graduates of the School Psychology program would be a valuable resource to support the mental health services provided in schools. However, there is no status data that reports the number of graduates from this program thus far or their placement status after graduation. Even with a well-coordinated utilization of all mental health professionals, Ethiopia could still encounter a substantial lack of mental health professionals. The shortage of mental health professionals would be even higher in providing services to children and adolescents since 46.5% of the Ethiopian population is under the age of 14 (https://data.un.org/en/iso/et.html).

Due to low number of adequately trained mental health professionals, Ethiopia largely depends on health extension workers to integrate mental health services into their prevention and intervention work on physical health. “Health extension workers are Ethiopia’s version of a large-scale community health workers (CHWs) program” (Assefa et al., 2019: 2). Health extension workers are members of a community (grade 12 graduates–sometimes with some health training experience) who are chosen by their respective district health service leaders to provide basic health and medical care to their community. However, these workers do not receive the relevant training required to assess, diagnose and intervene with individuals struggling with mental health problems. Ahmed et al. (2019: 6) indicated that a majority of urban health extension workers did not have the scientific knowledge of mental disorders and held unfavorable attitudes toward individuals with mental disorders. Most of these professionals perceived people with mental disorders as dangerous, unpredictable and better off in an institutionalized setting. In the same study, it was found that a majority of professional health extension workers believed mental disorders are curable and counseling and medication would be helpful. Although this is a single study, it clearly shows the dilemma health extension workers are facing. On one hand, they appear to accept cultural myths as causation for mental disorders just like any member of a community. On the other hand, they appear to believe in evidence-based interventions as a means of supporting individuals with mental health issues. This conflict, perhaps experienced by others in Ethiopia, results in individuals not receiving culturally responsive intervention training to address mental health issues and to promote overall wellness.

FMOH, in collaboration with the WHO, has been providing mental health Gap Action Program (mhGAP) trainings to health extension workers, in an attempt to address the lack of trained mental health professionals. The mhGAP is intended to equip primary care providers and their trainers with a model guide to mental health disorders, knowledge and skill on assessment, diagnosis, and intervention (World Health Organization, 2016a: 3; World Health Organization, 2016b). The training for health extension workers has been identified to be significantly improving their knowledge, attitude and skill. These trainings are aimed toward equipping health care providers with the information and skill that is required to work with individuals at the grassroots level (Faregh et al., 2019: 7). The training manual includes 5–6 days of basic mental health training so the primary care professionals can integrate knowledge of mental, neurological and substance use disorders into their service and reporting. In an Ethiopian context, there have been a significant number of health extension workers who have been trained with mhGAP training. The mhGAP version 2.0 Training manual for training of trainers (field testing version) specifies the length of training for each major area included in the manual (World Health Organization, 2016a: 61). For example, the length of training for child and adolescent mental and behavioral disorders is 5.8 h of which 3 h are allocated for assessment and management of mental health disorders (2 h on assessment and 1 h on management of mental health disorders), depression is 4.5 h and self-harm/suicide training is 3.75 h (World Health Organization, 2016a: 249). Comparatively, university programs across the world that train mental health professionals range anywhere from 2 to 8 years, and typically are degree-granting programs. While any training is better than none, it is clear that 16 h of training in child/adolescent mental health-related issues does not equate to a degree that prepares professionals to recognize symptoms, and to provide culturally responsive intervention strategies.

Despite the mhGAP training’s brief duration, several findings support the significant improvement in the knowledge, attitude and skill of mental health professionals after the training. However, studies conducted among mental health professionals indicated the perceived challenges in the implementation of culturally sound evidence-based therapeutic techniques. Cultural factors were identified as a challenge in the training of mhGAP for mental health professionals. In a reflection study of 6 years of experience in implementing the mhGAP training in six developing countries including Ethiopia, a study by Faregh et al. (2019: 4) identified that mental health professionals share some cultural beliefs with their community such as a stigma toward individuals with mental disorders. This study also explored mental health professionals’ beliefs that the cause of mental health problems was due to spiritual causes (e.g., curses) or the person’s responsibility. Moreover, the authors suggested that trainees may not have gained the clarity on how to translate and apply what they had learned in their mhGAP training into their practice. The study suggests that trainees may face real-life situations without the preparation and knowledge about comorbidity of mental and physical symptoms, as this is not well addressed in the training.

In a recent study that explored the indigenizing of westernized therapeutic techniques for Ethiopian communities, Wondie and Abawa (2019: 9) found that mental health professionals identified a challenge in adapting the theoretical concepts they learned through formal education to practical implementation. Participants in this study identified cultural perceptions of the causes of mental health problems (associating the cause of schizophrenia to spiritual powers), and perceived severity of mental health problems (minimizing anxiety and depression) and, therefore limiting the transferability of western-based therapeutic techniques to Ethiopians. Most absent from their knowledge-base were culturally tested techniques that would add to their ability to respond effectively to cases they are presented with. Another study by Zeleke et al. (2019: 226) supported the finding that cultural beliefs among community members is one of the greatest challenges of mental health workers. The authors identified that mental health professionals believed they needed more training on providing mental health services. Zeleke et al. (2019: 217) also pointed out the dominance of the medical approach as the primary model for teaching mental health professionals in Ethiopia. This in turn leads to the use of diagnostic-based approaches to address mental health issues. Participants in both studies identified the knowledge and skill gap, as well as a lack of culturally appropriate counseling skills to help their clients.

The medical approach or assessment-oriented mental health approach is prominent in the curricula of both undergraduate and graduate programs in psychology in Ethiopia. Since program curricula, especially for undergraduate programs, is harmonized across the country, all graduates learn using very similar course syllabi. For instance, the undergraduate psychology program, although it does include courses on child and adolescent development, testing and practicums, it lacks the integration of a relationship-focused wellness-oriented approach to mental health interventions. In the graduate level programs in clinical and social psychology, Counseling I and Counseling II courses emphasize the role of relationship between the counselor and the client. However, these courses do not include either a wellness approach or an emphasis on the relevance and intersection of self-care and informal community care, as recommended in the WHO pyramid model of comprehensive mental health services. Moreover, the practical work requirement (e.g., clinical psychology graduate program at one university) requires more hours for presenting diagnostic cases than designing interventions. This indicates the emphasis on identifying the psychopathology and becoming equipped with assessment tools and diagnoses based on Western diagnostic criteria, i.e., *Diagnostic and Statistical Manual of Mental Disorders,* fifth edition (DSM-5, 2013) published by the American Psychological Association while the intervention is given less focus. Although the *DSM-5* provides guidance in diagnosing mental disorders, the limited representation of cultures like Ethiopia in the process of conducting research to identify symptoms for a particular disorder puts Ethiopian professionals in a difficult position. For instance, the general perception of some symptoms of depression and anxiety among community members as acceptable reactions to life circumstances was one of the challenges identified by mental health professions in their effort to diagnose mental health disorders and deliver interventions for individuals (Wondie and Abawa, 2019: 8). Similar challenges can be experienced by providers using the *International Classification of Diseases and Related Health Problems 10th Revision* (World Health Organization, 2016) since there is no known culturally sensitive language translation and symptoms interpretation manual for use by Ethiopian mental health professionals. The lack of culturally sensitive and contextualized tools may present a challenge to mental health professionals when they face real-life practical cases that require translating what they learned in class into diagnosis and treatment planning. Practicing health professionals are also challenged with accessing supportive resources for themselves when faced with difficult cases that they feel unprepared or presented with less resources to manage.

Mentorship and supervision are one of the most reliable ways to strengthen the skills of early career professionals as they develop the necessary knowledge, attitude and skill to work effectively. Due to the lack of appropriately trained mental health professionals, Ethiopia also faces a challenge of having a system for supervising front line workers. Feregh et al. (2019: 6) identified the lack of follow up and supervision procedures that could enhance the implementation of mhGAP training knowledge, attitude and skill. The study specifically indicates that, in Ethiopian health care systems, mental health trained professionals did not have the training to supervise primary health care providers, contributing to the absence of an existing supervision system for health care workers in their practice of diagnosis and therapeutic intervention for their clients. With self-care at the foundation of the WHO pyramid model, it makes sense to emphasize the self-care needs of Ethiopian children and families as a method of bridging the gap between professional mental health training and culturally responsive mental health interventions. Perhaps the wellness model (Myers and Sweeney, 2004: 234–235; Myers and Sweeney, 2008: 483) could provide health care workers with practical tools to assist in their intervention efforts with children and families, further developing culturally responsive interventions and promoting the overall wellness of Ethiopians (Myers and Sweeney, 2008: 487–488).

### Bridging the Gap: The Wellness Model of Mental Health

**FIGURE 3:**
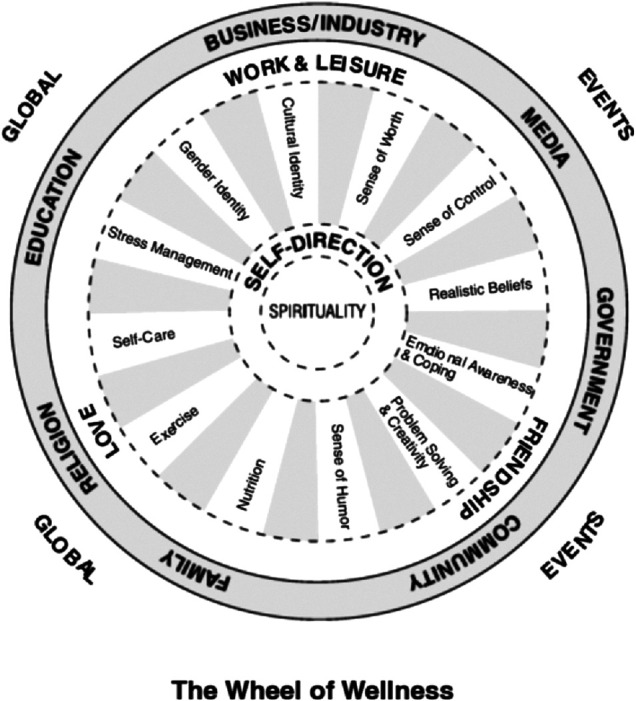
The wheel of wellness.

In the 1980s Myers and Sweeney developed the Wheel of Wellness, based on the Individual Psychology of Adler [(1927) 1954]. The Wheel of Wellness integrated concepts that contribute to holistic and healthy living, quality of life, and longevity. The Wheel of Wellness arose from Adler’s life tasks of work, friendship, love, and later self and spirit (Mosak and Dreikurs, 1967, as cited in Myers and Sweeney, 2004: 235). The Wheel of Wellness, and later the model of the Indivisible Self, take into account diversity and cultural considerations that make it applicable to many difficult cultural contexts. Figure 3 is a depiction of the original Wheel of Wellness developed by Witmer et al. (1998).

At the center of the wheel is spirituality, which in most cultural contexts is considered the most significant aspect of overall wellness. In an Ethiopian context, as many studies indicated (e.g., Faregh et al., 2019; Wondie and Abawa, 2019; Zeleke et al., 2019), spiritual beliefs seem to guide individuals’ understanding of the causation and preferred interventions for mental disorders. Enabling mental health professionals/workers to recognize the relevance of spirituality in the intervention for mental disorders would help the individual to feel supported and validated.

Directly around spirituality is self-direction, associated with each spoke of the wheel surrounding it. Depending on cultural context, self-direction could include an orientation toward family and community that influences an individual’s self-direction, if part of a collectivist culture as opposed to an individualist culture. Each spoke of the wheel represents tasks associated with achieving balanced wellness and mental health—cultural identity, sense of worth, sense of control, realistic beliefs, emotional awareness and coping, problem solving and creativity, sense of humor, exercise, self-care, stress management and gender identity (Myers and Sweeney, 2004: 236). It is reasonable to assume that the spokes of the wheel are relevant at certain points in time, some more relevant than others. While the ideal would be to achieve a balance of wellness in each spoke of the wheel, it is more likely that attention shifts to different aspects of wellness as needed and in response to mental health issues that present. Each task surrounding self-direction is oriented toward achieving Adler’s tasks of work/leisure, friendship, and love.

**FIGURE 4:**
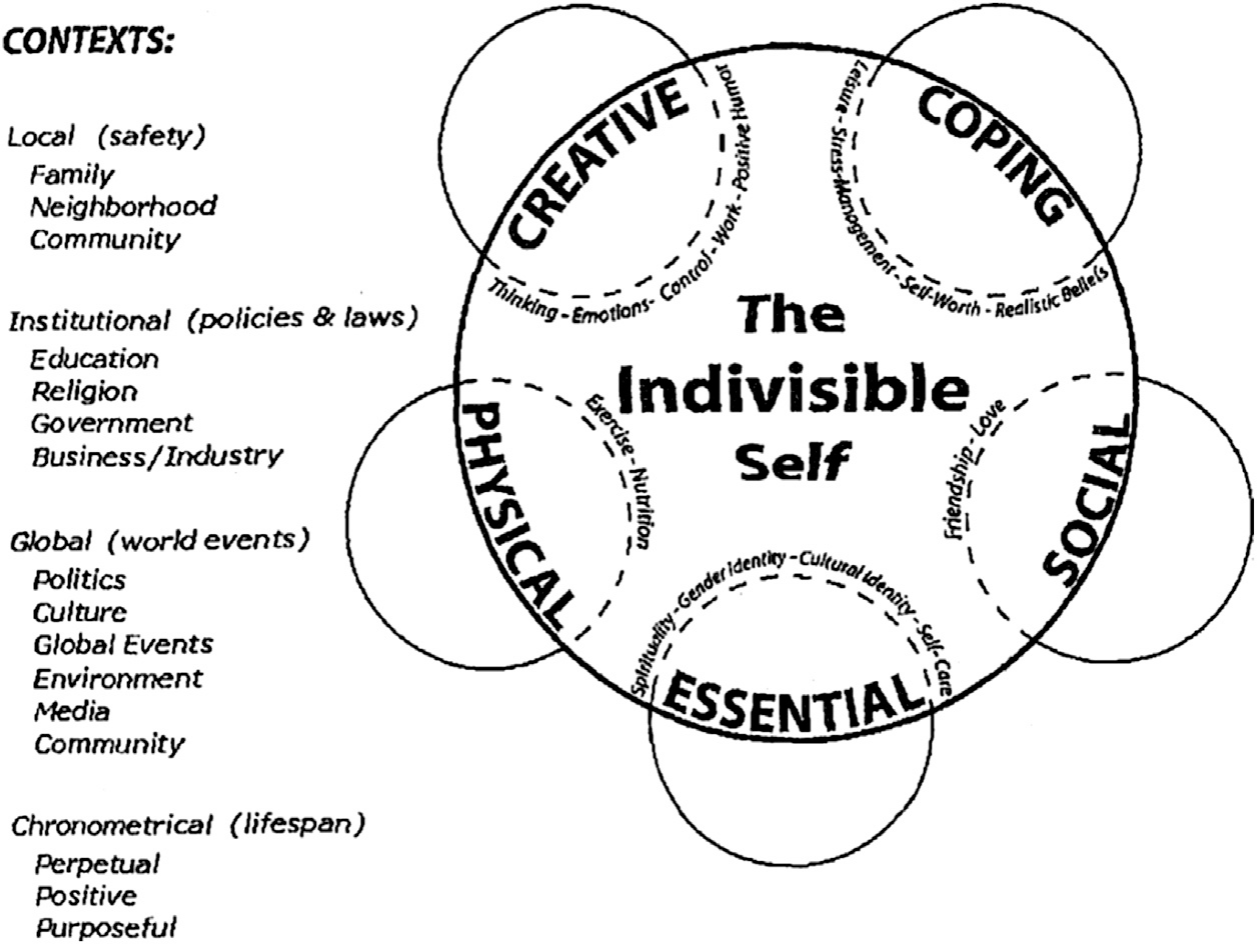
The indivisible self model.

Life forces that affect personal wellness, such as religion, family, education, business/industry, media, government and community surround the individual in the wheel of wellness, and on a larger scale, global issues and current events are addressed as contributing to overall wellness (Myers and Sweeney, 2004: 236). Further research revealed a better representation of overall wellness, pictured below in Figure 4: The Indivisible Self evidence-based model of wellness (Myers and Sweeney, 2004, 236; Myers and Sweeney, 2008, 484).

The Indivisible Self model includes all of the components of the original Wheel of Wellness, but reduces specific factors influencing overall wellness into more conceptual factors that encompass the many tasks formerly associated with the Wheel of Wellness. In addition, the new model acknowledges cultural contexts, as well as developmental life stages. This new model is accessible to individuals at all stages of the lifespan, in all cultural contexts (Myers and Sweeney, 2004, 239–240; Myers and Sweeney, 2008, 486).

The Indivisible Self model represents a framework that can be used to describe the somewhat elusive concept of self-care that the WHO established as the foundation for positive mental health. Not only does the model break down the concept of self-care into specific factors that encompass the whole of human experience, but it brings the focus of attention to strengths an individual, and arguably, family and community system possesses, as opposed to a sole focus on illness and psychopathology. Familiarity with the Indivisible Self model could provide healthcare workers in Ethiopia with a framework to assess mental health issues presenting in their clients, and a direction for providing culturally responsive interventions. Following are some suggestions about how orientation to the Wellness and Indivisible Self models could facilitate services provided to children in Ethiopia.

### On-The-Job Training for School-Based Professionals

A significant percentage of children and adolescents spend majority of their daytime in schools. For instance, the Ethiopian Ministry of Education stated that the Net Enrollment Ratio (NER) for primary school children (7–14 years-olds) was 94.7% (Federal Ministry of Education, 2019: 24 and 41). This data presents a relevant fact that mental health services involving children and adolescents can be addressed in school settings in addition to formal mental health facilities. Desta et al. (2017: 34) looked at the possibility of integrating mental health interventions, mainly the screening of mental health disorders in the early childhood care services, as a means to provide access to children. The study found that early childhood care and education teachers benefited from trainings on early identification of mental disorders. The authors suggested that schools can support the formal health system stranded by a lack of mental health professionals by identifying cases early and providing care and support. Using the Indivisible Self model (Myers and Sweeney, 2004, 237; Myers and Sweeney, 2008, 484) as a reference in considering what sources of care and support a child might need, school personnel can assess what strengths a child and family already possess—in what areas a child is doing well, and what areas could use more attention. From a wellness, strengths-based perspective, building upon strengths can often alleviate challenges presenting in other areas. Perhaps a child has unrecognized strengths that they can apply to specific problems they are facing, or assistance can be provided to families/communities in managing symptoms that are presenting as problematic. While knowledge in diagnosing mental disorders is an important aspect of the formal training of mental health professionals, it does not always equate to culturally responsive interventions. Perhaps orienting mental health workers and other human services personnel to the Indivisible Self model could allow for culturally responsive intervention without the need for diagnostic assessment, especially when human service professionals are not adequately trained to do so.

### Adoption of Child/Adolescent Specific Mental Health Initiatives

Despite the high prevalence of mental health problems among children in Ethiopia, the FMOH does not have a specific plan or strategy to address mental health issues in children and/or adolescents (World Health Organization, 2017a: 1). Considering the developmental milestones children and adolescents go through as they transition to the next stages of their lives, mental health services should be specifically designed to address their needs. The establishment of developmental milestone-specific mental health strategies would allow the country to develop initiatives and action plans to build a system for children/adolescents to achieve overall wellness. Developing a national strategy would open a platform for training of professionals in child/adolescent specific mental health issues and/or general mental health trainings. The Indivisible Self model could bridge the gap between traditional university training in diagnosis and assessment of mental disorders, the WHO’s pyramid model of mental health, and the Ethiopian cultural orientation toward spiritual connection.

The development of a national strategy would also allow collaboration with mental health professionals, although few, that are already working with children and adolescents in schools. These professionals include applied psychology, school psychology, and social psychology graduates to be counted as mental health service providers. Furthermore, it would allow schools to adopt the strategy and design accountable action plans to promote the overall wellness of their students.

## Discussion

The design of mental health professionals’ training curricula should be culturally sensitive to enable trainees to translate their training into practice. It is imperative that international frameworks for assessment, diagnosis and intervention are implemented in accordance with the local contexts of Ethiopian culture. For instance, although using the *DSM-5* as a reference to diagnose mental disorders is valuable, it is paramount that professionals learn about the cultural considerations for each diagnosis. In this regard, Wondie and Abawa’s (2019: 11) study among mental health professionals in Ethiopia found that the conceptualization and intervention of mental health disorders is perceived differently than in Western cultures mostly because of cultural factors. In addition, the translation of mental disorder symptoms in a local language without a guiding document poses a challenge for mental health professionals. Therefore, trainees need to be equipped with the knowledge and the skill in identifying culturally specific symptoms that are presenting as problematic to the person in need. Familiarity with the Indivisible Self model could enable professionals to design intervention strategies that go hand-in-hand with the values of the person they are helping; acknowledging their strengths and challenges.

Introducing a relationship-based wellness model as opposed to the diagnosis-focused medical model would alleviate some of the concerns raised above. Ethiopians believe in establishing and maintaining close relationships with each other. Most people have shared values and consistently work hard to maintain the equilibrium of this status quo. Based on the recommendations of the WHO pyramid model’s mix of mental health services framework, professional helpers should recognize individuals’ self-care strategies that are derived from their values and preferences. Further, they should design interventions that integrate the informal community in to their mental health recovery.

Effective integration of the Indivisible Self model to the education and training of mental health professionals can be done in multiple phases. First, continuing education or in-service programs could provide information about overall wellness and the Indivisible Self model to professionals providing services to children and families who were not specifically trained in mental health. Integrating basic knowledge about holistic wellness, recognizing strengths, and enlisting support from families and communities could help to alleviate symptoms that children present. Second, the curriculum design of university programs could be tailored toward promoting the self-care of the individual seeking mental health services. Integrating topics, courses, and experiential learning tasks to the curriculum could enable mental health professionals in training to coordinate the formal and informal systems, self-care, and professional mental health services that their future clients require. These strategies could enable both professionals and the immediate community members to accept the individual with mental health issues as a person first and not as a diagnosis. Third, working with schools, neighborhoods and parents could be an effective strategy to work toward comprehensive mental health services that integrate informal and formal sectors. Developing a system of accountability in schools would also encourage the school system to establish and/or strengthen the mental health systems within schools. Finally, establishing a mentoring and supervision system could provide an opportunity for mental health workers to consult on culturally specific challenges in the process of assessment, diagnosis and intervention for mental disorders among children and adolescents. This mentoring and supervision platform could create an opportunity for integrating and developing culturally responsive strength-based strategies that are supported by the lived experiences of Ethiopian children and adolescents.

## Conclusion

The potential implementation of the above proposed interventions could enhance the access and quality of mental health services for Ethiopians, especially children and adolescents. Introducing a system that acknowledges the relevance of continuing education will help mental health professionals to be familiar with adaptable contemporary mental health interventions. Consultation among professionals is key to generating ideas that could solve challenges experienced by mental health professionals. It creates the opportunity for psychologists, social workers, and counselors to work across disciplines which might lead to collaborative evidence-based research focusing on culturally sensitive mental health interventions. This systematic review also brings to light the need for ongoing research on the implementation and outcome of the Indivisible Self model specifically within an Ethiopian context. College level mental health programs could pilot introducing the concept and implementation of the wellness approach with the goal of integrating culturally sensitive concepts and interventions into the overall approach to mental health care. On the bases of its applicability, future policies and strategies could integrate the Indivisible Self model in to the WHO’s pyramid model of optimal mix of mental health services.

Improving access to culturally relevant intervention strategies to better serve children and adolescents in Ethiopia is at the heart of our purpose in this paper. Systemic change takes time, and perhaps the best place to start is to acknowledge the need for an inclusive and culturally responsive approach to mental health care. Acknowledgment could lead to continuing education for direct care (health extension) workers, followed by improved collaboration between health extension workers and mental health professionals. If preliminary outcome data support the inclusion of a wellness approach to mental health care with children and families, perhaps that will provide the impetus for including curricula and practice implementing the wellness approach and the Indivisible Self model into higher education training programs. Promoting the holistic health and wellness of children and adolescents in Ethiopia, and around the world, facilitates wellness in families, communities, and larger social contexts. The world is calling for change, and perhaps honoring cultural contexts in our approach to mental health care is but one way to answer the call.

## Author Contributions

HM is the lead author for this manuscript. HM and VJ consulted on organization and outline and divided tasks. HM conducted literature review on mental health approaches and services in Ethiopia. VJ provided context on the wellness and indivisible self models. Authors worked together to edit and integrate contents in the manuscript.

## Conflict of Interest

The authors declare that the research was conducted in the absence of any commercial or financial relationships that could be construed as a potential conflict of interest.
